# The large Hellenic Study of Uveitis: epidemiology, etiologic factors and classification

**DOI:** 10.1007/s10792-023-02772-5

**Published:** 2023-07-10

**Authors:** Dimitrios Kalogeropoulos, Ioannis Asproudis, Maria Stefaniotou, Marilita M. Moschos, Vassilios P. Kozobolis, Paraskevi V. Voulgari, Andreas Katsanos, Constantina Gartzonika, Chris Kalogeropoulos

**Affiliations:** 1 Department of Ophthalmology, Faculty of Medicine, School of Health Sciences, University of Ioannina, University Campus, 451 10 Ioannina, Greece; 2 1st Department of Ophthalmology, General Hospital of Athens G. Gennimatas, Medical School, National and Kapodistrian University of Athens, Athens, Greece; 3 Department of Ophthalmology, Faculty of Medicine, School of Health Sciences, University of Patras, Patras, Greece; 4 Department of Rheumatology, Faculty of Medicine, School of Health Sciences, University of Ioannina, Ioannina, Greece; 5 Laboratory of Microbiology, Faculty of Medicine, School of Health Sciences, University of Ioannina, Ioannina, Greece

**Keywords:** Uveitis, Infectious, Non-infectious, Epidemiology, Demography

## Abstract

**Purpose:**

To analyse the demography, etiology, and classification of uveitis at a tertiary academic referral center.

**Methods:**

An observational study was conducted on the archives of uveitic patients at the Ocular Inflammation Service of the Department of Ophthalmology at the University Hospital of Ioannina (Greece) from 1991 to 2020. This study aimed to investigate the epidemiological profile of patients, including their demographics and the main etiologic factors of uveitis.

**Results:**

Out of 6191 cases with uveitis, 1925 were infectious, 4125 were non-infectious, and an overall of 141 masquerade syndromes were recorded. Among these cases, 5950 patients were adults, with a slight female predominance, while 241 were children (< 18 years old). Interestingly, 24.2% of cases (1500 patients) were associated with 4 specific microorganisms. Herpetic uveitis (HSV-1 and VZV/HZV) was the most common cause of infectious uveitis (14.87%), followed by toxoplasmosis (6.6%) and tuberculosis (2.74%). In 49.2% of non-infectious uveitis cases, no systematic correlation was found. The most frequent causes of non-infectious uveitis included sarcoidosis, white dot syndromes, ankylosing spondylitis, lens-induced uveitis, Adamantiades-Behçet disease, and idiopathic juvenile arthritis. Infectious uveitis was more common in the rural population, whereas non-infectious uveitis was more frequently recorded in the urban population

**Conclusions:**

Although our study was conducted on a predominantly white Caucasian population, it also reflects the effect of increasing immigration, improvements of diagnostic techniques, changes in referral patterns, and various actual changes in disease incidence.

## Introduction

Uveitis comprises a very diverse group of vision-threatening intraocular inflammatory conditions attributed to a wide spectrum of etiologies and pathogenetic mechanisms [1]. Technically, the term uveitis encompasses inflammation of the uveal tract, which comprises the iris, ciliary body and choroid. The inflammatory process may also involve the surrounding tissues, such as the retina (retinitis), optic disc (papillitis) and vitreous (vitritis) [2]. Uveitis can cause a significant burden of legal and economic blindness worldwide, particularly among the working-age population [1, 2]. Additionally, it may be the first manifestation of a severe systemic infectious or non-infectious disorder, serving as a critical warning for a meticulous diagnostic work-up and prompt medical intervention [2]. The causal association with a vast number of systemic pathologies, including autoimmune disorders, rheumatic diseases, masquerade syndromes, and systemic infections, highlights the clinical importance of a multidisciplinary approach in the management of uveitic patients.

The incidence of uveitis in the United States and Europe is estimated to be approximately 20–50/100,000 per year, and prevalence is estimated to be in the order of 38-714/100000 population [3, 4]. The phenotypic expression and prevalence of different types of uveitis depend on age, gender, race, geographic distribution, genetic factors, environmental influence, socioeconomic status and social habits [4, 5].

Recording patterns of uveitis within a population is essential for understanding its epidemiological profile, which may be useful for improving clinical practices. Accurate etiological and anatomical classification of uveitis contributes decisively to the differential diagnostic approach, improved understanding of the pathogenetic mechanisms, and development of novel therapeutic approaches. Our study is the first major epidemiological approach of uveitis in Greece over the last 30 years and one of the largest single-center uveitis studies in the literature to date. A second study presents diagnostic and therapeutic algorithms, complications, and final outcome [6].

## Material and methods

The data for this study was obtained from a doctoral dissertation conducted at the Department of Ophthalmology in the University Hospital of Ioannina, Greece. Our analysis covers a long observational period of 30 years, from 1991 to 2020, and includes patients who were examined until the end of 2020 and monitored until the end of the first semester of 2021. We collected and analysed data retrospectively with special emphasis on clinical findings, diagnostic exploration, therapy, and outcome. The research protocol was approved by the Scientific Board of the Faculty of Medicine (School of Health Sciences) of the University of Ioannina (Greece) (846^α^/27-3-2018), and followed the tenets of the Declaration of Helsinki.

The patients referred to our service come from the entire prefecture of Ioannina and the surrounding areas of Epirus, including Thesprotia, Arta, and Preveza, as well as Etoloakarnania, Western Macedonia, and the Ionian Islands, with a focus on Corfu and Lefkada. Since our Service is an officially established center for the study of uveitis, it also receives referrals from all over Greece, Cyprus, and Albania. This is the largest study concerning the analysis of uveitis in Greece in one of the largest public centers in the country, and one of the largest studies worldwide.

The first part of our analysis aims to record the rates of etiologic factors in patients with uveitis, providing a better understanding of the epidemiology and demographics, as well as how these factors are influenced by other factors such as immigration. The second part of our analysis focuses on diagnostic and therapeutic approaches, describing the factors that have led to improved holistic management of uveitis. Specifically, our database included the following information:

- The epidemiological and demographic characteristics: gender, age, nationality, race, place of origin and residence, social and travel history, and occupation.
- The findings of the ophthalmological examination
- History of ophthalmic diseases.
- History and findings from the systemic clinical examination
- Laboratory results.
- The treatment: topical treatment, systemic treatment, or a combination of them. Surgical treatment.
- The course of the disease: cure, relapses, and complications.

Moreover, we evaluated the afore-mentioned data in order:

- To classify uveitis as infectious, non-infectious, and masquerade syndromes, based on the current literature. To achieve this, two different types of classification were used with the ultimate goal of the specific and clear classification of the various pathological entities associated with uveitis. *Classification A* is based on the guidelines of the International Ocular Inflammation Society (IOIS) and the approach of Foster and Vitale [7]. On the other hand, *Classification B* follows the guidelines of the SUN (Standardization of Uveitis Nomenclature) Group, the International Uveitis Study Group (IUSG), and the approach of A. Brézin [8]. Both classifications classify uveitis as infectious, non-infectious and masquerade syndromes. The main difference between *classification A* and *B* is that in the latter (*B*), chronic postoperative endophthalmitis and endogenous endophthalmitis are included in the infectious causes of uveitis rather than as masquerade syndromes. Even in Nussenblatt & Whitcup's classic book [9], there is no clear position on where these entities belong. In our study, *classification A* was preferred.
- To record the causes of infectious uveitis and their frequency over time, including a breakdown of frequencies every five years. It is worth noting that our center did not classify post-traumatic bacterial and fungal endophthalmitis, as well as acute postoperative septic endophthalmitis, as uveitis due to their distinct pathogenetic features compared to other causes of infectious uveitis.
- To classify non-infectious uveitis into two categories: those with a known associated systemic disease and those without a known associated systemic disease (either initially or during the follow-up period), and to quantify and record their frequency (similarly to infectious uveitis). Cases in which the cause of uveitis could not be identified were classified as idiopathic.
- To record masquerade syndromes and describe their differential approach.
- To describe the anatomical classification (anterior, intermediate, posterior, and panuveitis) of uveitis concerning:
- The differential diagnostic approach
- Treatment
- The outcome of uveitis
- To record the number of patients with uveitis (examined for the first time) every five years.

Further analysis of the diagnostic exploration and therapeutic approach will be thoroughly provided in a second paper [6].

## Results

From January 1st, 1991, to December 31st, 2020, we examined 6191 patients with uveitis in our center. The study excluded patients who only had scleritis without uveitis and those with an underlying systemic disease, such as sarcoidosis, juvenile idiopathic arthritis (JIA), Adamantiades-Behçet disease (ABD), etc., but without ocular involvement, who were referred for routine control. Our study includes patients who were investigated for an episode of uveitis, those with a known associated systemic disease, and patients referred by other clinicians (from the public or private sector), with or without a known diagnosis, to receive specialized healthcare services. The majority of patients received complete management (diagnosis and/or treatment) at the University Hospital of Ioannina and were referred to other specialties (e.g., rheumatologists, gastroenterologists, paediatricians) when necessary. After completing the required diagnostic exploration and treatment, a smaller percentage of patients returned for long-term follow-up at the center or with the ophthalmologist from whom they were initially referred, or returned to their region of origin. Some of these patients returned to our center for further examinations, as well as other medical procedures, such as surgical treatment of uveitis-associated complications. The follow-up period for 5673 cases ranged from 6 months to 18 years, while for 518 cases (approximately 8.4%), it was less than 6 months. Out of the total of 6191 cases, the mean follow-up period was 5.3 ± 2.5 years.

Table 1 summarizes the patients' origin by geographical area, highlighting the increasing volume of referrals from different regions. While the majority of patients (2400 cases) were from Ioannina and the rest of Epirus (1031 cases), a significant number of patients came from other parts of Greece, including Northern Greece, Western Greece, Central Greece, Peloponnese, and the Ionian Islands. Outside of Greece, a significant number of patients (385) were from Albania, while a relatively smaller number of uveitis cases came from other geographic areas.

**Table 1:** Number of patients examined in the Ocular Inflammation Service (1991–2020) by geographical area

Geographical area	Number of patients
Prefecture of Ioannina	2400
Rest of Epirus	1031
Corfu	392
Lefkada	120
Etoloakarnania	313
North west και central Macedonia	424
Eastern Macedonia and Thrace	199
Thessaly	140
Attica	201
Peloponnese	30
Rest of Greece	489
Cyprus	24
Albania	385
Asia	11 (India-Pakistan: 8, Middle East: 2, East Asia: 1)
Latin America	2 (Brazil)
Africa	2 (Sudan: 1, Egypt: 1)
Republic of North Macedonia	2
Europe	26 (Tourists, professionals or immigrants)
Total number of patients	6191

Comments. The prefecture of Ioannina and the rest of Epirus, Corfu, Lefkada and Etoloakarnania are defined as *region A* of the Greek territory and the other regions of the Greek territory as *region B*

Out of the 6191 cases, 3291 were female patients (F) and 2900 were male patients (M) (F:M = 1.13:1). Among them, 5950 were adults (age ≥ 18 years), with 3175 women and 2775 men (proportion of female to male adults = 1.14:1). The remaining 241 cases were children with uveitis, including 125 males and 116 females (proportion of male to female children = 1.07:1). The age of patients ranged from 1 day to 88 years (x̄ ± SD = 40.6 ± 19.1 years).

**Fig. 1:**
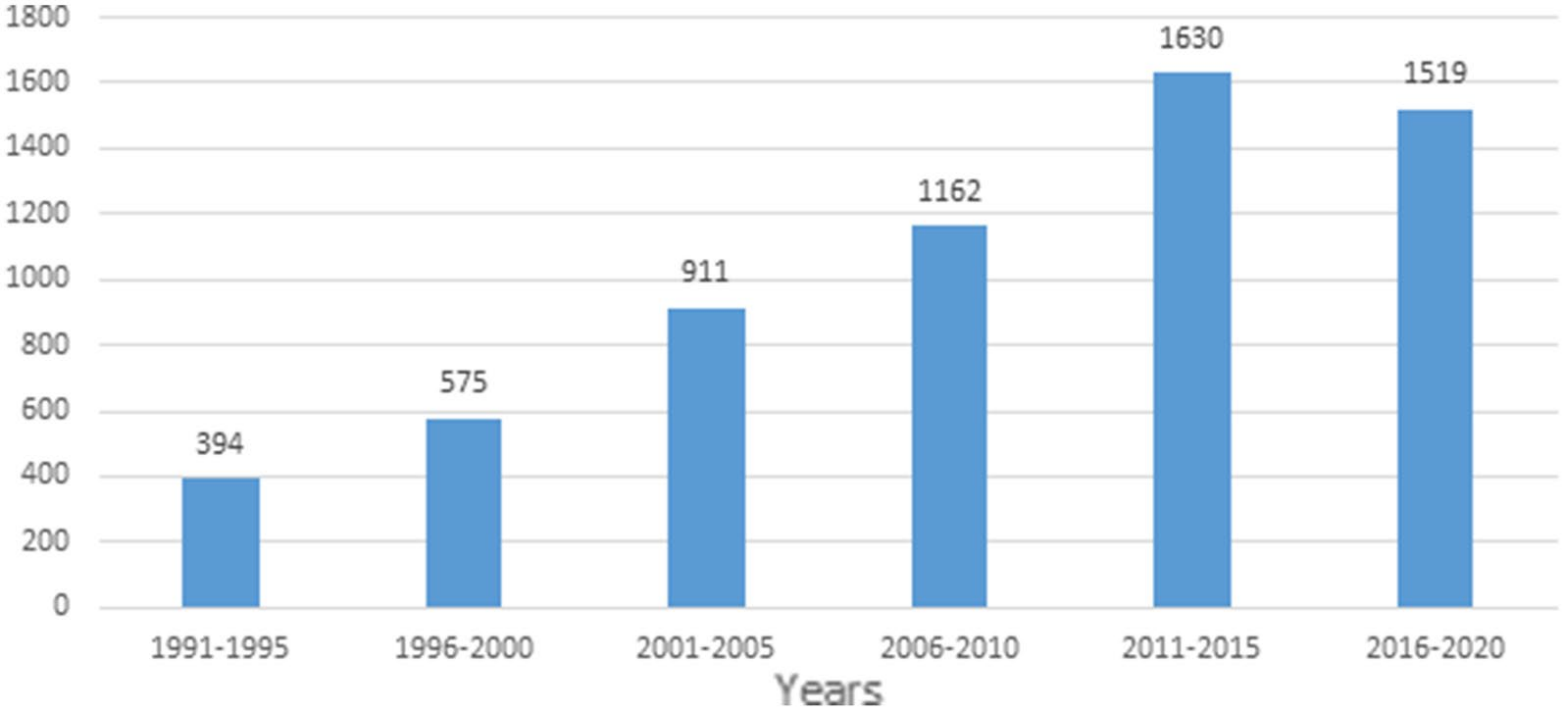
This chart illustrates the number of patients diagnosed with uveitis every five years, indicating a gradual increase over the study period. The number of uveitis cases in the period of 1991–1995 was 394, and this was followed by a significant exponential increase, which continued until the period of 2011–2015, where it reached 1630 cases. In the period of 2016–2020, the number of patients decreased to 1519, which is most likely attributed to the Covid-19 pandemic

Figure 1 displays the number of uveitis patients in five-year intervals, illustrating a gradual increase over the study period. The number of patients with uveitis from 1991 to 1995 was 394, followed by a significant exponential increase until 2011–2015, reaching 1630 cases. However, the number of patients decreased to 1519 in 2016–2020, which appears to be largely related to the Covid-19 pandemic.

Based on *Classification A*, there were 1925 cases of infectious uveitis, 4125 cases of non-infectious uveitis, and 141 cases of masquerade syndromes. Excluding masquerade syndromes, 31.8% of cases were caused by infectious agents while 68.2% were due to non-infectious causes.

Table 2 provides a detailed description of the causes of infectious uveitis, including the total number of patients (including children) for each disease entity/cause listed separately. Viruses are identified as the primary cause of infectious uveitis (1162), followed by parasites (442) and bacteria (321), accounting for 60.40%, 22.90%, and 16.70% of infectious uveitis cases, respectively. Notably, an interesting pattern was observed regarding the incidence of the two major imitators, syphilis and tuberculosis. Specifically, tuberculous uveitis gradually increased during the first fifteen years of our study, but declined in the following fifteen years. Conversely, syphilis (and other spirochetes) was rarely detected in the first ten years, but demonstrated a significant elevation in the subsequent twenty years.

**Table 2:** Infectious uveitis

Etiologic factor	Total number of patients	Children (aged < 18 years-old)
**Bacteria**	321	2
*Mycobacterium tuberculosis*	170	
*Spirochaete*	64 (*Treponema pallidum*: 23, *Leptospira*: 30, *Borrelia burgdorferi*: 11)	
*Bartonella* spp.	25	
*Brucella* spp.	56	2
*Mycobacterium leprae*	1	
*Tropheryma whipplei*	2	
*Rickettsia* spp.	2	
Meningo-uveal syndrome	1	
**Parasites**	442	8
*Toxoplasma gondii*	409	5
*Toxocara canis*	31	3
*Dirofilaria*	2	
**Viruses**	1162	19
Herpes viruses	1003	8
**Herpetic uveitis** HSV-1 HSV-2 VZV (HZO) CMV EBV	986 654 20 259 32 21	8
Acute retinal necrosis (ARN)* HSV-1 HSV-2 VZV (HZO) CMV	11 5 2 3 1	
CMV & HIV retinitis*	2	
CMV in immunocompromised patients*	4	
*Coxsackie viruses*	73	3
*Echoviruses*	38	2
*Adenoviruses*	5	
HIV retinitis	2	
*Measles morbillivirus*	2	2
*Rubella virus*	2	2
HAV	3	
HBV	2	
HCV	1	
Influenza viruses	29	5
Presumed viral uveitis	2	

*ARN* acute retinal necrosis, *CMV* cytomegalovirus, *EBV* Epstein-Barr virus, *HAV*: hepatitis A virus, *HBV* hepatitis B virus, *HCV* hepatitis C virus, *HIV* human immunodeficiency virus, *HSV* herpes simplex virus, *HZO* herpes Zoster ophthalmicus, *VZV* Varicella–Zoster virus

Comments: *These cases do not correspond to herpetic uveitis, while they constitute distinct clinical entities, and authors decided to present them separately from herpetic uveitis

Infectious uveitis was found to occur across a wider age range and was diagnosed more frequently in men (M:F = 1.8:1). Among children, the most common causes of infectious uveitis were attributed to viruses (19 patients in total) and parasites (8 patients in total).

Interestingly, four microorganisms are responsible for the largest number of infectious uveitis cases. Specifically, herpetic uveitis is the most common cause of infectious uveitis in the population studied. Among all patients with uveitis, 10.64% (659 patients) were due to Herpes Simplex Virus 1 (HSV-1) and 4.23% (262 patients) were due to Varicella Zoster Virus (VZV). Following herpesviruses in frequency, toxoplasmosis accounted for 6.6% (409 patients) of cases, and tuberculosis accounted for 2.74% (170 patients). Therefore, 24.2% of all uveitis cases (1500 patients) are attributed to these four microorganisms.

Out of the total 4125 cases of non-infectious uveitis, which represents the largest percentage of cases in our center, 2096 were attributed to a known systemic disease. Among them, in 1027 cases (29.30%), the diagnosis was already established at the time of the initial presentation, while in the remaining 889 cases (21.50%), the diagnosis was made during the diagnostic workup for uveitis or at a later stage during the patient's follow-up. In 2029 cases (49.20%), there was no discernible systemic association. It is worth noting that in more than one-fifth of non-infectious uveitis cases, the eye was the first or even the only organ affected.

Table 3 provides a breakdown of the total number of patients (including children) for each disease entity associated with non-infectious uveitis. Among the patients studied, sarcoidosis (348 patients) was found to be the most common pathology associated with uveitis, followed by white dot syndromes or other non-systemic diseases that present with white dots (332), ankylosing spondylitis (158), ABD (131 patients), phacoanaphylactic uveitis (141 patients), and JIA (121 patients). Therefore, this group of non-infectious uveitis includes uveitis with a specific clinical appearance, such as white dot syndromes, which represent distinct pathological entities. Specifically, as shown in Table 3, White dot syndromes comprise a total of 10 distinct clinical entities, which explains their relatively large number compared to other causes.

**Table 3:** Non-infectious uveitis associated with a known systemic disease (including non-infectious uveitis with specific clinical appearance)

Etiologic factor	Total number of patients	Children (aged < 18 years-old)
I. Seronegative Spondyloarthropathies		
1. Ankylosing spondylitis	158	
2. Reactive arthritis*	19	
3. Psoriatic arthritis	32	
II. Inflammatory bowel disease (IBD) (± arthritis)		
1. Crohn's disease	26	1
2. Ulcerative colitis	27	
III. HLA-B27 (without systemic associations)	17	
IV. Juvenile idiopathic arthritis (JIA)	121	121
V. Nephritis		
1. TINU syndrome	14	
2. Glomerulonephritis	4	1
VI. Major systemic syndromes		
1. Sarcoidosis	348	2
2. Adamantiades-Behçet disease	131	1
3. Vogt-Koyanagi-Harada (VKH) syndrome	10	
VII. Major syndromes with retinal vasculitis		
1. Polyarteritis Nodosa	12	
2. Systemic lupus erythematosus (SLE)	9	
3. Granulomatosis with polyangiitis (GPA) (previously known as Wegener’s disease)	9	
4. Giant cell arteritis (temporal arteritis) (GCA)	6	
5. Polymyalgia rheumatica	2	
6. Scleroderma	2	
7. CREST syndrome	2	
8. Relapsing polychondritis	1	
9. Buerger's disease (thromboangiitis obliterans)	1	
VIII. Sclero-uveitis (in autoimmune diseases)	30	
IX. Multiple sclerosis	67	7
X. White dot syndromes**		
1. MEWDS	38	
2. APMPEE	29	
3. PIC	33	
4. DMC	7	
5. ARPE	5	
6. Multifocal choroiditis with vitritis	42	
7. Birdshot chorioretinopathy (BCR)	30	
8. Serpiginous choroiditis	41	
9. Ampiginous choroiditis	1	
10. Multifocal choroiditis with panuveitis	106	
XI. Uveitis attributed to viral-induced immune deviation		
1. Glaucomatocyclitic Crisis (Posner-Schlossman Syndrome)	44	
2. Fuchs heterochromic iridocyclitis	78	
XII. Uveitis associated with endocrine diseases		
1. Diabetes Mellitus	4	
2. Hypothyroidism	7	
XIII. Reactive uveitis		
1. Sinusitis	62	
2. Tonsillitis (Including post-streptococcal)	48	
3. Dental inflammation	81	
4. Otitis media	2	2
5. Enteritis (*Yersinia* spp., *Shigella* spp., *Salmonella* spp., viruses)	42	
6. *Helicobacter pylori*	7	
7. Acute Macular Neuroretinopathy (AMNR)	1	
XIV. Other uveitis		
1. Sympathetic ophthalmia	33	
2. Phacoanaphylactic uveitis	141	
3. Traumatic uveitis (non-penetrating trauma)	65	
XV. Distinct entities with retinal vasculitis		
1. Eales Disease	25	
2. Acute multifocal hemorrhagic retinal vasculitis	2	
3. IRVAN	1	
XVI. Drug-induced uveitis	72	

*AMNR* acute macular neuroretinopathy, *ARPE* acute retinal pigment epitheliitis, *APMPEE* acute posterior multifocal placoid pigment epitheliopathy, *BCR* Birdshot chorioretinopathy, *CREST* calcinosis, Raynaud's phenomenon, esophageal dysmotility, sclerodactyly, and telangiectasia, *DMC* Discrete multifocal choroiditis, *GCA* giant cell arteritis, *GPA* granulomatosis with polyangiitis, *HLA* human leukocyte antigen, *MEWDS* multiple evanescent white dot syndrome, *IRVAN* Idiopathic retinal vasculitis-aneurysms-neuroretinitis syndrome, *PIC* punctate inner choroidopathy, *SLE* systemic lupus erythematosus, *TINU* tubulointerstitial nephritis and uveitis syndrome, *VKH* Vogt–Koyanagi–Harada

^*^Reactive arthritis: previously known as Reiter's syndrome

^**^White dot syndromes or non-systemic diseases presenting with white dots

Reactive uveitis refers to a special category in which an antigenic stimulus, not necessarily microbial, triggers uveitis indirectly through immune mechanisms. The stimulus can originate in the adjacent anatomical area to the eye or in a remote area of the body.

Non-infectious uveitis, with or without a systemic association, was more common in patients younger than 55 years (92%) and 60 years (95%), respectively, with a slightly higher prevalence in females (M:F = 1:1.2 and M:F = 1:1.7, respectively).

**Fig. 2:**
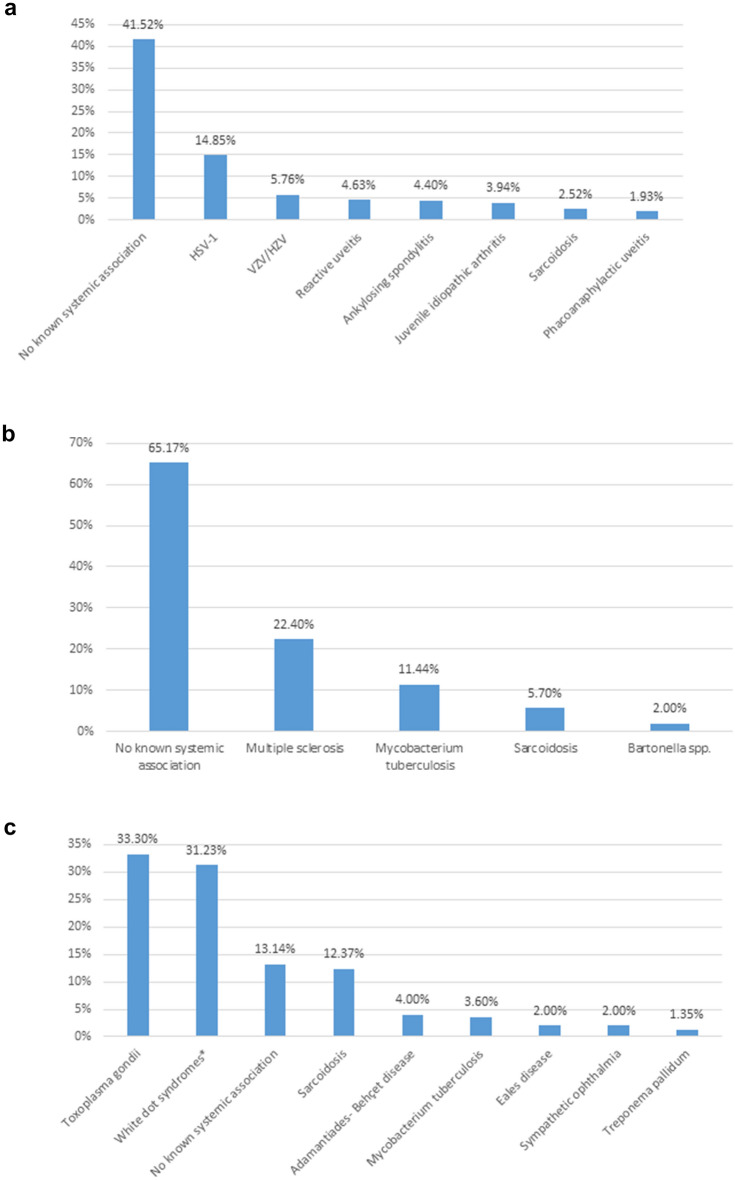

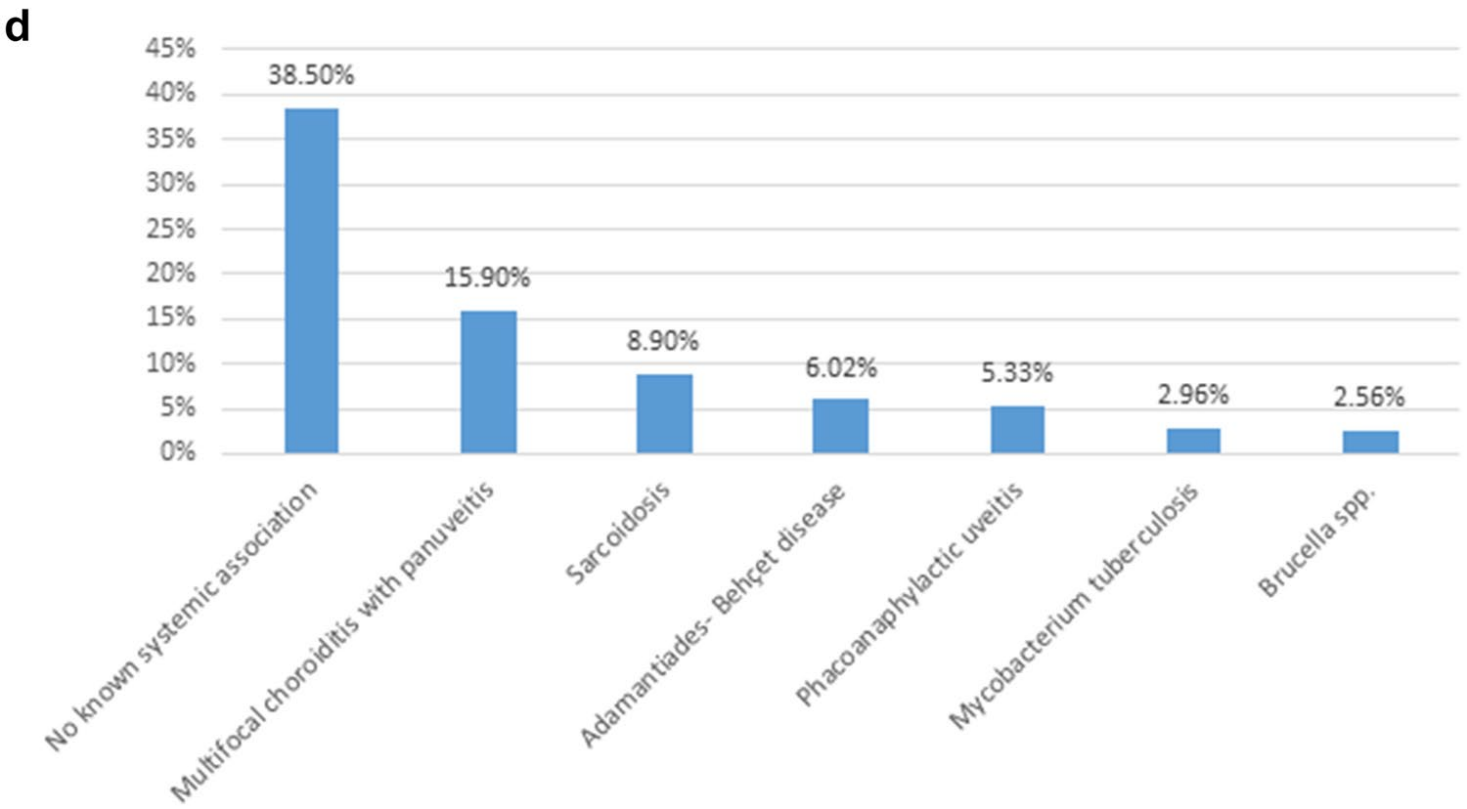
The anatomical classification of uveitis (anterior, intermediate, posterior, and panuveitis) is summarized in Tables 5 and 6, as well as in Fig. 2 (a-d) for each entity separately. As seen in Table 3, White Dot Syndromes comprise a total of 10 distinct clinical entities, which explains their relatively large number compared to other causes. *White dot syndromes or clinical entities presenting with white dots

Consequently, the results of our study indicate that the 10 most common causes of uveitis include HSV-1, VZV / HZV, *Toxoplasma gondii*, *Mycobacterium tuberculosis,* sarcoidosis, white tot syndromes, phacoanaphylactic uveitis, ankylosing spondylitis, ABD and JIA. These etiological factors collectively account for 44.1% (2731 out of a total of 6191 cases) of all uveitis cases in our center. Again, as displayed in Table 3 and Fig. 2c, white dot syndromes encompass a total of 10 distinct clinical entities, which accounts for their relatively high prevalence compared to other common causes of uveitis.

Masquerade syndromes represent a unique category of pathologies characterized by clinical findings that complicate the differential diagnostic approach, requiring a thorough investigation not only by ophthalmologists but also by physicians from other specialties. The cases classified as masquerade syndromes account for 2.27% (141 cases) of our cases. Among them, 58 cases (0.93%) were attributed to neoplastic causes, 66 cases (1.06%) to endophthalmitis, and 17 cases (0.27%) to non-neoplastic and non-infectious etiologies.

Respectively, Table 4 describes in detail the number of patients with masquerade syndromes. These pathologies include malignant diseases (e.g., intraocular lymphomas or paraneoplastic syndromes), endophthalmitis (late-onset or endogenous), and benign non-infectious diseases (e.g., retinitis pigmentosa or pigment dispersion syndrome). Masquerade syndromes were observed almost equally among the two genders (M:F = 1:1.1) and mostly in patients older than 58 years (83%).

**Table 4:** Masquerade syndromes (classification Α in our study)

Malignant diseases	
Intraocular lymphomas	15
Leukemias	8
Metastatic carcinomas	12
Paraneoplastic syndromes • Associated with carcinomas • Associated with melanomas • Associated with multiple myeloma	23 15 6 2
**Endophthalmitis**	
***Chronic post-operative endophthalmitis (late-onset)***	
*Coagulase Negative Staphylococcus (CNS)*	3
*Propionibacterium acnes*	16
***Endogenous endophthalmitis***	
Bacteria	
• *Staphylococci* • *Streptococcus viridans* • *Escherichia coli* • *Proteus mirabilis* • *Serratia marcescens* • *Citrobacter koseri* • *Haemophilus parainfluenzae* • *Acinetobacter baumannii*	12 2 11 3 2 1 1 1
Fungi	
• *Candida albicans* • *Aspergillus* spp. • *Cryptococcus* spp. • *Sporothrix schenkii* • *Coccidioides* spp.	8 3 1 1 1
**Non-malignant, non-infectious diseases**	
Rhegmatogenous retinal detachment	4
Retinitis pigmentosa	2
Intravitreal foreign body	1
Pigment dispersion syndrome	5
Ocular Ischemic Syndrome	4
Amyloidosis	1

The anatomical classification of uveitis (anterior, intermediate, posterior, and panuveitis) is summarized in Tables 5 and 6, as well as in Fig. 2a–d for each entity separately. In Tables 5 and 6, the number of eyes is written in parentheses next to the number of patients because in bilateral uveitis the affected or diseased anatomical area may be different between the two eyes.

**Table 5:** Infectious uveitis

	Number of patients (Number of eyes)			
Etiological factor	Anterior uveitis	Intermediate uveitis	Posterior uveitis	Panuveitis
**Bacteria**				
*Mycobacterium tuberculosis*	65 (70)	38 (46)	47 (61)	30 (30)
*Treponema pallidum*	–	2 (3)	16 (23)	5 (8)
*Leptospira* spp.	10 (14)	4 (5)	14 (17)	2 (2)
*Borrelia* spp.	3 (5)	3 (4)	3 (4)	2 (2)
*Bartonella* spp.	3 (4)	8 (8)	12 (12)	2 (2)
*Brucella* spp.	36 (40)	–	–	20 (26)
*Mycobacterium leprae*	1 (1)	–	–	–
*Tropheryma whipplei*	–	–	–	2 (2)
*Rickettsia* spp.	2 (3)	–	–	–
Bacterial meningo-uveal syndromes	–	–	–	1 (1)
**Parasites**				
*Toxoplasma gondii*	–	–	395 (565)	14 (16)
*Toxocara canis*	–	3 (4)	15 (15)	13 (15)
*Dirofilaria*	–	2 (2)	–	–
**Viruses**				
HSV-1	654 (670)	–	–	5 (5)
HSV-2	20 (20)	–	–	2 (2)
VZV (HZO)	251 (260)	2 (4)	4 (4)	5 (5)
EBV	15 (17)	–	–	–
CMV	30 (32)	–	–	2 (3)
Coxsackie viruses	66 (70)	1 (2)	1 (1)	2 (2)
Echoviruses	33 (39)	1 (2)	1 (1)	1 (1)
Adenoviruses	5 (6)	–	–	–
Measles morbillivirus	2 (4)	–	–	–
*Rubella virus*	2 (3)	–	–	–
HAV	3 (4)	–	–	–
HBV	2 (3)	–	1 (1)	–
HCV	–		1 (1)	
Influenza viruses	24 (40)	–	–	5 (5)
Viral meningo-uveal syndromes	–	–	–	2 (2)
HIV retinitis	–	–	2 (4)	–
CMV (in HIV patients)	–	–	2 (3)	–
CMV (in immunosuppressed)	–	–	3 (4)	1 (1)

*CMV* cytomegalovirus, *EBV* Epstein–Barr virus, *HAV* hepatitis A virus, *HBV* hepatitis B virus, *HCV* hepatitis C virus, *HIV* human immunodeficiency virus, *HSV* herpes simplex virus, *HZO* Herpes Zoster Ophthalmicus, *VZV* Varicella-zoster virus

**Table 6:** Non-infectious uveitis

	Number of patients (number of eyes)			
Etiologic factor	Anterior uveitis	Intermediate uveitis	Posterior uveitis	Panuveitis
Ι. Seronegative Spondyloarthropathy				
1. Ankylosing spondylitis	158 (199)	–	–	–
2. Reactive arthritis*	19 (20)	–	–	–
3. Psoriatic arthritis	32 (35)	–	–	–
II. Inflammatory bowel disease (IBD) (± arthritis)				
1. Crohn's disease	26 (31)	–	–	–
2. Ulcerative colitis	27 (34)	–	–	–
III. HLA-B27 (without systemic associations)	17 (19)	–	–	–
IV. Juvenile idiopathic arthritis (JIA)	121 (178)	–	–	–
V. Nephritis				
1. TINU syndrome	14 (20)	–	–	–
2. Glomerulonephritis	4 (7)	–	–	–
VI. Major systemic syndromes				
1. Sarcoidosis	86 (114)	17 (23)	181 (210)	64 (90)
2. Adamantiades-Behçet disease	34 (40)	3 (4)	53 (68)	42 (61)
3. Vogt-Koyanagi-Harada (VKH) syndrome	–	–	1 (2)	9 (18)
VII. Major syndromes with retinal vasculitis				
1. Polyarteritis Nodosa	3 (4)	–	6 (9)	3 (5)
2. Systemic lupus erythematosus (SLE)	–	–	7 (11)	2 (3)
3. Granulomatosis with polyangiitis (GPA) (previously known as Wegener’s disease)	1 (1)	1 (2)	4 (7)	3 (4)
4. Giant cell arteritis (temporal arteritis) (GCA)	4 (4)	–	–	–
5. Polymyalgia rheumatica	2 (3)	–	–	–
6. Scleroderma	2 (3)	–	–	–
7. CREST syndrome	1 (1)	1 (1)	–	–
8. Relapsing polychondritis	1 (2)	–	–	–
9. Buerger's disease (thromboangiitis obliterans)	1 (2)	–	–	–
VIII. Sclero-uveitis (in autoimmune diseases)	26 (38)	–	4 (6)	–
IX. Multiple sclerosis	–	67 (89)	–	–
X. White dot syndromes**				
1. MEWDS	–	–	38 (55)	–
2. APMPEE	–	–	29 (58)	–
3. PIC	–	–	33 (41)	–
4. DMC	–	–	7 (10)	–
5. ARPE	–	–	5 (7)	–
6. Multifocal choroiditis with vitritis	–	–	42 (60)	–
7. Birdshot chorioretinopathy (BCR)	–	–	30 (60)	–
8. Serpiginous choroiditis	–	–	41 (77)	–
9. Ampiginous choroiditis	–	–	1 (1)	
10. Multifocal choroiditis with panuveitis	–	–	–	106 (161)
XI. Uveitis attributed to viral-induced immune deviation				
1. Glaucomatocyclitic Crisis (Posner-Schlossman Syndrome)	44 (50)	–	–	–
2. Fuchs heterochromic iridocyclitis	78 (82)	–	–	–
XII. Uveitis associated with endocrine diseases				
1. Diabetes mellitus	4 (6)	–	–	–
2. Hypothyroidism	7 (12)	–	–	–
XIII. Reactive uveitis				
1. Sinusitis	50 (51)	–	–	12 (12)
2. Tonsillitis (Including post–streptococcal)	40 (46)	–	–	8 (8)
3. Dental inflammation	70 (75)	–	–	11 (11)
4. Otitis media	2 (2)	–	–	–
5. Enteritis (*Yersinia* spp., *Shigella* spp., *Salmonella* spp., viruses)	29 (30)	–	–	13 (14)
6. *Helicobacter pylori*	7 (7)	–	–	–
7. Acute Macular Neuroretinopathy (AMNR)	–	–	1 (1)	–
XIV. Other uveitis				
1. Sympathetic ophthalmia	–	–	17 (34)	16 (32)
2. Phacoanaphylactic uveitis	87 (87)	–	–	54 (54)
3. Traumatic uveitis (non-penetrating trauma)	65 (65)			
XV. Distinct entities with retinal vasculitis				
1. Eales Disease	–	–	25 (34)	–
2. Acute multifocal hemorrhagic retinal vasculitis	–	–	2 (3)	–
3. IRVAN	–	–	1 (2)	–
XVI. Drug-induced uveitis	60 (90)	–	1 (2)	11 (12)
**Non-infectious uveitis with no known systemic associations or ophthalmic pathologies that do not correspond in distinct uveitic entities**	1338 (1892)	203 (262)	180 (223)	308 (390)

*AMNR* acute macular neuroretinopathy, *ARPE* acute retinal pigment epitheliitis, *APMPEE* acute posterior multifocal placoid pigment epitheliopathy, *BCR* Birdshot chorioretinopathy, *CREST* calcinosis, Raynaud's phenomenon, esophageal dysmotility, sclerodactyly, and telangiectasia, *DMC* discrete multifocal choroiditis, *GCA* giant cell arteritis, *GPA* granulomatosis with polyangiitis, *HLA* human leukocyte antigen, *MEWDS* multiple evanescent white dot syndrome, *IRVAN* Idiopathic retinal vasculitis-aneurysms-neuroretinitis syndrome, *PIC* punctate inner choroidopathy, *SLE* systemic lupus erythematosus, *TINU* tubulointerstitial nephritis and uveitis syndrome, *VKH* Vogt–Koyanagi–Harada

*Reactive arthritis: previously known as Reiter's syndrome

**White dot syndromes or non-systemic diseases presenting with white dots

Examining the patients' files in the department's archive reveals a gradual improvement in obtaining and completing the medical history over the decades. Therefore, although the initial overall rate of uveitic cases without a definite diagnosis was 32.77%, this figure declined gradually over time (when comparing the first and last 5-year intervals of our study), reflecting the improvement in the diagnostic approach to uveitis.

Based on Table 5 and 6, the anatomical distribution of uveitis in 6050 patients (excluding 141 masquerade syndromes) showed that 4557 eyes (59.1% of the total) had anterior uveitis, 461 eyes (5.99%) had intermediate uveitis, 1685 eyes (21.85%) had posterior uveitis, and 1007 eyes (13.06%) had panuveitis. Of the cases with anterior uveitis, 28.63% were due to infectious causes, while 71.37% were non-infectious. For intermediate uveitis, 17.35% of the eyes were caused by infectious agents, and 82.65% were non-infectious. In posterior uveitis, 41.8% of cases were caused by infectious agents, while 58.2% were non-infectious. Finally, for eyes with panuveitis, 12.9% were caused by infectious agents, while 87.1% were non-infectious uveitis.

A correlation is made between the anatomical classification and the cause of uveitis, to conclude the anatomical area that tends to be inflamed in the most common pathological entities Fig. 2a–d. More specifically, anterior uveitis, with a significant difference from other entities of known etiology, is mainly caused by herpes viruses. Nevertheless, in a large percentage of cases with anterior uveitis, there is no etiological correlation (Fig. 2a). In most patients with intermediate uveitis (Fig. 2b) no systematic association is finally recognized, while the known causes include multiple sclerosis, tuberculosis, sarcoidosis, and *Bartonella* spp. infection (which has been also considered a significant imitator in our study) (Table 5). In posterior uveitis (Fig. 2c) the three most common causes are *Toxoplasma gondii*, white dot syndromes (or non-systemic clinical entities presenting with white dots), uveitis with an unknown systemic association, and sarcoidosis. Finally, in cases with panuveitis (Fig. 2d), uveitis with no systemic association predominates (also known as idiopathic uveitis), followed by multifocal choroiditis with panuveitis, sarcoidosis, ABD, and phacoanaphylactic uveitis.

**Fig. 3:**
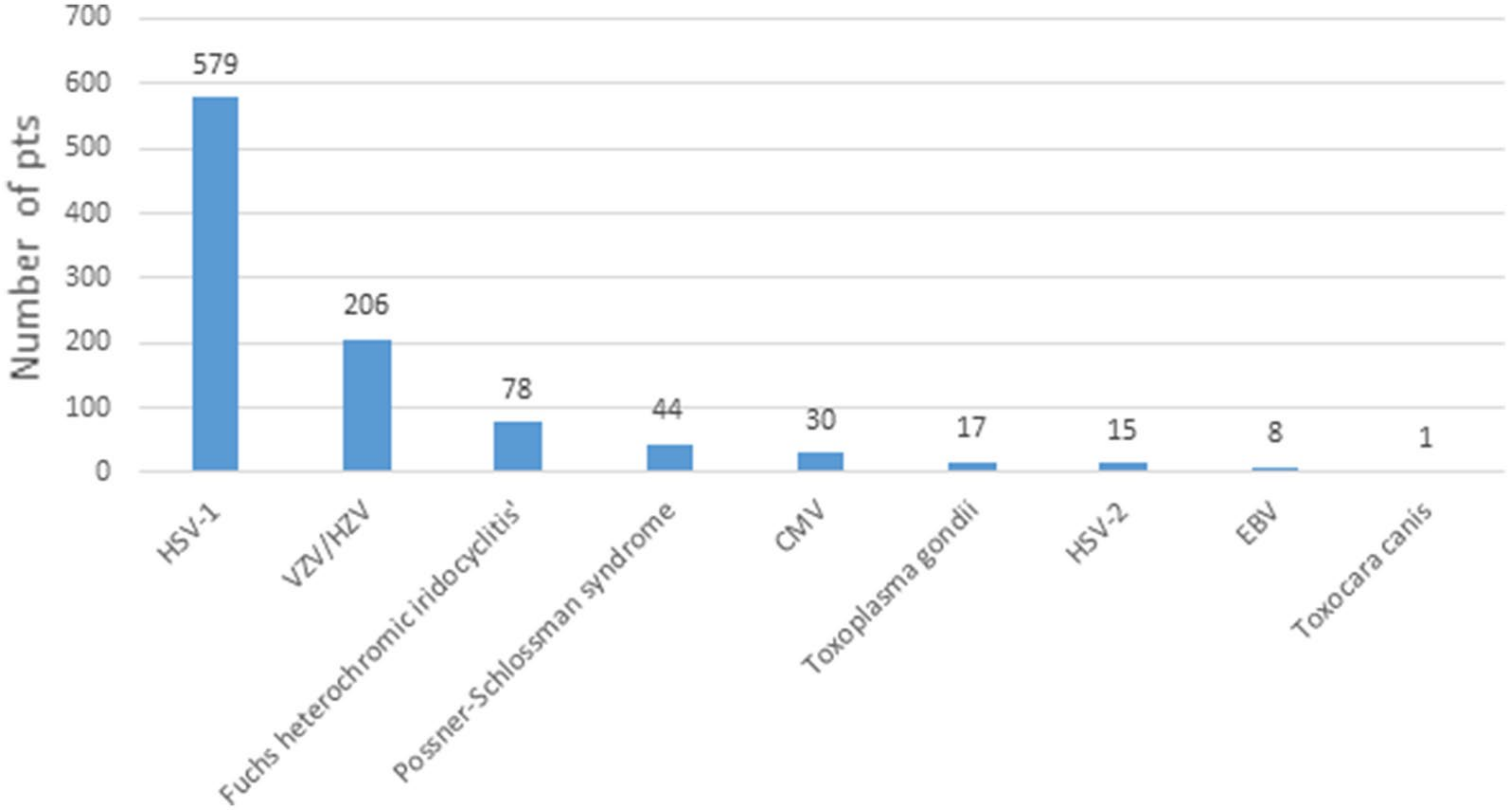
As seen in this figure, which summarizes the most common causes of hypertensive uveitis, 85.68% of hypertensive uveitis (838 out of 978) is attributed to herpes viruses, mainly HSV-1 and VZV / HZV. In patients with uveitis due to HSV-1 and VZV, hypertensive uveitis occurred in 87.43% and 78.64% of them, respectively. *HSV*: herpes simplex virus, *HZV*: herpes zoster virus, *VZV*: Varicella zoster virus

Hypertensive uveitis is a common and clinically significant manifestation of uveitis in the studied patient population, with 15.8% of uveitis cases (978 out of 6191 cases) presenting with hypertensive uveitis. Figure 3 summarizes the most common causes of hypertensive uveitis, with 85.68% (838 out of 978 cases) attributed to herpes viruses, mainly HSV-1 and VZV/HZV. In patients with uveitis caused by HSV-1 and VZV, hypertensive uveitis occurred in 87.43% and 78.64% of cases, respectively.

## Discussion

Ioannina is both the capital and the largest city of the Epirus region. The University Hospital of Ioannina provides a broad spectrum of medical services to a significant portion of the surrounding population and is one of the largest hospitals in the country. Notably, the Ocular Inflammation Service is the sole official academic department in Greece dedicated to the investigation of uveitis and ocular inflammation.

The ability to access medical care is not solely determined by geographical location, but is also influenced by demographic and socio-economic factors.

To our knowledge, this study presents the largest number of uveitic patients (6191 cases) examined in a referral center that specializes in ocular inflammations and infections. Furthermore, the study's duration of 30 years makes it the longest work conducted in the field of uveitis. As anticipated, our findings, when compared to those of other referral centers worldwide [10–28], exhibit notable variances in the number of patients diagnosed with specific ailments (e.g., Vogt-Koyanagi-Harada syndrome, which is rare in Greece). This disparity is linked to various factors, including geographical distribution, socio-economic criteria, environmental factors, genetic predisposition, and others. Additionally, increased migration and population mobility, travel, and globalization have already begun to impact the epidemiology of various diseases. As a result, we expect further modifications in the profile of these diseases within each studied population.

Our cases originate not only from the district of Epirus but also from other parts of the country and abroad. Over the 30 years of this study, we observed a gradual increase in the number of cases from outside the prefecture of Ioannina, as well as a general rise in referrals from other centers and more remote areas. This suggests that the experience of a specialized center, as well as the gradual improvement in the treatment and management of uveitis patients, has contributed to the increase in cases. Despite this upward trend, we noted a slight decline in the number of uveitic patients in the last year, which can be attributed to the Covid-19 pandemic.

A slight female predominance was observed, and the age range of the patients ranged from 1 day to 88 years (mean ± SD = 40.6 ± 19.1 years). Infectious causes of uveitis were observed across a wider range of ages, in contrast to non-infectious uveitis, which is more commonly attributed to a known systemic association or distinct clinical entity and is more prevalent in individuals under 55 years of age. The highest percentage of patients with uveitis without a known systemic correlation were younger than 60 years, while the rate of suspicion for masquerade syndrome was higher in patients over 58 years old.

Our study used *Classification A* to analyze a total of 6191 cases of uveitis, of which 1925 were infectious, 4125 were non-infectious, and 141 were masquerade syndromes. Over the 30-year study period, the frequency of certain diagnoses has changed. This observation is attributed to fluctuations in the incidence of uveitis-associated diseases such as tuberculosis or syphilis, as seen in the results. Specifically, in the studied population (as shown in Table 2), viruses were the most common infectious cause, followed by parasites and bacteria.

It is noteworthy that approximately 24.2% of uveitis cases were attributed to four causes: HSV-1, VZV/HZV, *Toxoplasma gondii, and Mycobacterium tuberculosis*. In contrast, no associations with systemic diseases or other distinct entities were found in 49.2% of non-infectious uveitis cases. Interestingly, in more than one-fifth of non-infectious uveitis cases, the eye was the first or only target organ, indicating the need for a thorough investigation of uveitic patients suspected of underlying systemic disease (e.g., sarcoidosis, ankylosing spondylitis). Finally, although masquerade syndromes constituted only 2.27% of our cases, their diagnosis and identification can be particularly critical for the patient's life, as malignancy may be underlying. It is important to note that taking a detailed and careful medical history plays a prominent role in the study of uveitis. As suggested by our results, the gradual improvement in obtaining and completing medical histories was one of the most critical parameters that contributed to the reduction of cases without a definite diagnosis.

Infectious uveitis is characterized by a significant prevalence in the developing world, representing 30–50% of all uveitis [1, 4, 5, 29]. Relevant studies report that infectious uveitis most often manifests as posterior uveitis and panuveitis in these areas. The most common infectious causes include tuberculosis, toxoplasmosis, onchocerciasis, cysticercosis, herpetic uveitis, leptospirosis, leprosy, and other various parasitic diseases [4, 30]. The prevalence of these clinical entities varies depending on the geographical area. In studies from Africa and South America, infectious uveitis accounts for about one-third of all uveitis cases, with toxoplasmosis being the most common causative agent [31, 32]. In Saudi Arabia, infectious uveitis is responsible for 36% of cases, with the most common cause being herpetic anterior uveitis (16%) [33]. A relatively recent nationwide survey from China, indicated that the most common infectious causes of uveitis in China are syphilis, human immunodeficiency virus (HIV), HSV, VZV and tuberculosis [28]. In India, infectious uveitis is responsible for 30.7% of all cases, with leptospirosis and tuberculosis being the most common causative agents [1, 5, 18]. In developed countries, infectious uveitis is responsible for a significantly smaller proportion of cases. The most common causes of infectious uveitis in the Western world include toxoplasmosis and herpetic uveitis [10, 12, 34–36]. On the other hand, tuberculosis and syphilis are less common, with an overall lower prevalence (< 3%) [34]. However, an increase in the prevalence of tuberculosis has been reported in Japan [37, 38] and the Netherlands [35], an observation that contradicts our findings. Finally, patients from rural areas of Poland have a significantly higher incidence of infectious uveitis compared to other European countries [39], an observation that is in line with our results for rural populations. Recent studies support variation in the prevalence of infectious uveitis between different age groups in developing countries. Infectious uveitis usually seems to affect children more than in developed countries, an observation that comes in agreement with our results. Entities often associated with intraocular inflammation in childhood are parasitic anterior uveitis (29.6%), endophthalmitis (8%), leptospirosis (5.5%), and toxoplasmosis (4.7%). In the same areas, in the middle-aged, the common etiologies reported are leptospirosis (10.5%), tuberculosis (5.9%), and herpes (4.5%) [5]. Finally, in the elderly, the most common causes are herpes (12.1%), leprosy (3.6%), and leptospirosis (3.4%) [5, 40].

In our population, the majority of non-infectious etiologies (Table 6) cause anterior uveitis, except for sarcoidosis and ABD, which also occur in the form of posterior uveitis, and Vogt-Koyanagi-Harada (VKH) disease which causes panuveitis. However, in addition to VKH, panuveitis is also recorded in 18.4% of patients with sarcoidosis and 32% of patients with ABD. Finally, the typical presentation of uveitis related to white dot syndromes concerns the posterior segment. Non-infectious uveitis is generally more common in developed countries, with significant differences in their global distribution [5]. The most common causes of non-infectious uveitis include HLA-B27-related anterior uveitis (4–32%), Fuchs’ syndrome, sarcoidosis, Vogt-Koyanagi-Harada syndrome, sympathetic ophthalmia, birdshot chorioretinopathy, serpiginous choroiditis, and ABD [4]. Of particular interest is the fact that an infection may initially give the impression of non-infectious uveitis, raising concerns in the differential approach [41]. As mentioned, HLA-B27-associated anterior uveitis has been reported as one of the most common types of non-infectious uveitis in developed countries, except Japan (2.5%) [42] and Italy (2.4%) [14]. In our study, these cases accounted for 6.76% of non-infectious uveitis. The prevalence of Fuchs’ syndrome ranges from 1.8% to 22.7% in the developed world but does not exceed 0–5.6% in the developing world [4]. In China, India, Thailand, Iraq, and Saudi Arabia, Vogt-Koyanagi-Harada syndrome and sympathetic ophthalmia have been reported as relatively common causes of non-infectious uveitis [5, 19, 22, 43]. In addition, birdshot chorioretinopathy appears to be absent in the same populations, while it is more common in western countries [34]. Sarcoidosis is also a relatively common cause (5–18.1%) in the United States, Germany, the United Kingdom, the Netherlands, Switzerland, and Japan [10, 12, 25, 36, 44], but is less common in Italy (2.2%) [45], Israel (0.5%) [46], Portugal (1.6%) [14], and China (0.1%) [19]. In our center, sarcoidosis corresponds to 8.43% of non-infectious uveitis (Table 3). Serpiginous choroiditis has also been reported as a common cause of posterior uveitis. ABD is the leading cause of non-infectious uveitis in Turkey [15, 44]. It is also common in Saudi Arabia [33], Israel [46], China [19], Iran [20], Iraq [48], and Japan (6.5–32.2%) [35]. In the developed world, the most common non-infectious uveitis in children is JIA [40, 49], in contrast to traumatic uveitis and pars planitis in developing countries [15]. In adults, sarcoidosis (8–20%) and seronegative spondyloarthropathy (6–6.5%) are the most common causes of non-infectious uveitis in developed countries [50, 51], in contrast to phacogenic uveitis (mainly in the elderly) in developing countries. Regarding the category of non-infectious uveitis, in our material, white dot syndromes (or the clinical entities presenting with white dots) are the second known cause of non-infectious uveitis after sarcoidosis. This is largely due to the increased use of indocyanine green angiography (ICGA) in posterior uveitis, especially after 2004.

Reactive uveitis is a significant subset of non-infectious uveitis, which may arise due to an inflammatory lesion or disorder adjacent or distant to the eye, indicating a cascade of immunological deviation leading to uveitis. The diagnosis of this type of uveitis, with a specific cause such as chronic sinusitis, dental inflammation, or the presence of Helicobacter pylori, [52–54] has increased in our patient cohort, particularly in the last decade, which reflects the center's experience in the diagnostic approach to uveitis [6].

Masquerade syndromes pose a significant challenge in the field of intraocular inflammations, as they can mimic uveitis and mislead clinicians, resulting in severe consequences for the patient's vision, general health, and even their life [55]. Rothova et al. [56] reported the results of a study involving 1906 patients with intraocular inflammation. Of all patients initially diagnosed with intraocular inflammation, 116 (6%) had non-inflammatory causes, including neoplastic causes in 36/116 (31%) and non-neoplastic causes in 52/116 (45%). Additionally, 26 patients (22% and 1.4% of the total) were diagnosed with drug-induced uveitis, and 2 (2% and 0.1% of the total) had paraneoplastic uveitis. B-cell lymphoma was the most common neoplastic etiology, while non-neoplastic causes included vascular disorders (38%), hereditary retinal diseases (31%), and degenerative eye diseases (19%) [56]. Our results show that malignant causes accounted for 41.13% of masquerade syndromes (Table 4).

Our study reports an increase in the number of uveitis cases from areas outside our district over the last fifteen years, which reflects the growing experience of our center in treating uveitis. Documenting patients' origin is essential for understanding the epidemiology of different diseases and contributes to the diagnostic approach. Our results show that infectious uveitis is more common in rural populations, while non-infectious uveitis is more prevalent in urban areas. The ten most common causes of uveitis in our study are HSV-1, VZV/HZV, *Toxoplasma gondii*, and *Mycobacterium tuberculosis* for infectious uveitis, and sarcoidosis, white dot syndromes, phacoanaphylactic uveitis, ankylosing spondylitis, ABD, and JIA for non-infectious uveitis. We observed a gradual increase in herpes viruses and a decrease in cases of tuberculous uveitis in terms of infectious causes. Overall, non-infectious causes exhibited an increase in the number of patients with white dot syndrome (or non-systemic diseases presenting with white dots) and those with reactive uveitis, mainly due to the improved diagnostic accuracy of uveitis.

## Conclusions

This study presents the epidemiological results of the Ocular Inflammation Service at the University Hospital of Ioannina (Greece), which is the largest single-center survey in Greece and one of the largest worldwide, conducted by a specialist uveitis service. The study has analyzed the profile of uveitic patients over a period of 30 years in a tertiary academic referral center, comparing significant differences among various studies worldwide. However, such comparisons can be complex and challenging, and may not necessarily reflect accurate differences among studied populations. Therefore, further multicenter prospective studies are required to better understand the profile of uveitic patients in different geographic areas. A future paper from this center will present diagnostic and therapeutic algorithms, complications, and final outcomes.

## References

[1] de Smet MD, Taylor SR, Bodaghi B (2011). Understanding uveitis: the impact of research on visual outcomes. Prog Retin Eye Res.

[2] Krishna U, Ajanaku D, Denniston AK, Gkika T (2017). Uveitis: a sight-threatening disease which can impact all systems. Postgrad Med J.

[3] Durrani OM, Meads CA, Murray PI (2004). Uveitis: a potentially blinding disease. Ophthalmologica.

[4] Tsirouki T, Dastiridou A, Symeonidis C (2018). A focus on the epidemiology of uveitis. Ocul Immunol Inflamm.

[5] Rathinam SR, Namperumalsamy P (2007). Global variation and pattern changes in epidemiology of uveitis. Indian J Ophthalmol.

[6] Kalogeropoulos D, Asproudis I, Stefaniotou M (2023). The large Hellenic study of uveitis: diagnostic and therapeutic algorithms, complications, and final outcome. Asia Pac J Ophthalmol (Phila).

[7] Foster CS, Vitale AT (2013). Diagnosis and treatment of uveitis.

[8] Brézin AP, Batteux F (2010). Les uvéites.

[9] Whitcup SM, Sen HN (2021). Whitcup and Nussenblatt's Uveitis, E-Book: fundamentals and clinical practice.

[10] Jones NP (2015). The Manchester uveitis clinic: the first 3000 patients-epidemiology and casemix. Ocul Immunol Inflamm.

[11] Rodriguez A, Calonge M, Pedroza-Seres M (1996). Referral patterns of uveitis in a tertiary eye care center. Arch Ophthalmol.

[12] Jones NP (2015). The Manchester uveitis clinic: the first 3000 patients, 2: uveitis manifestations, complications, medical and surgical management. Ocul Immunol Inflamm.

[13] Palmares J, Coutinho MF, Castro-Correia J (1990). Uveitis in northern Portugal. Curr Eye Res.

[14] Mercanti A, Parolini B, Bonora A (2001). Epidemiology of endogenous uveitis in north-eastern Italy: analysis of 655 new cases. Acta Ophthalmol Scand.

[15] Merrill PT, Kim J, Cox TA (1997). Uveitis in the southeastern United States. Curr Eye Res.

[16] Tran VT, Auer C, Guex-Crosier Y (1994). Epidemiology of uveitis in Switzerland. Ocul Immunol Inflamm.

[17] Oruc S, Kaplan AD, Galen M, Kaplan HJ (2003). Uveitis referral pattern in a midwest university eye center. Ocul Immunol Inflamm.

[18] Singh R, Gupta V, Gupta A (2004). Pattern of uveitis in a referral eye clinic in north India. Indian J Ophthalmol.

[19] Yang P, Zhang Z, Zhou H (2005). Clinical patterns and characteristics of uveitis in a tertiary center for uveitis in China. Curr Eye Res.

[20] Soheilian M, Heidari K, Yazdani S (2004). Patterns of uveitis in a tertiary eye care center in Iran. Ocul Immunol Inflamm.

[21] Sengun A, Karadag R, Karakurt A (2005). Causes of uveitis in a referral hospital in Ankara. Turkey Ocul Immunol Inflamm.

[22] Kazokoglu H, Onal S, Tugal-Tutkun I (2008). Demographic and clinical features of uveitis in tertiary centers in Turkey. Ophthalmic Epidemiol.

[23] Sittivarakul W, Bhurayanontachai P, Ratanasukon M (2013). Pattern of uveitis in a university-based referral center in southern Thailand. Ocul Immunol Inflamm.

[24] Goto H, Mochizuki M, Yamaki K (2007). Epidemiological survey of intraocular inflammation in Japan. Jpn J Ophthalmol.

[25] Ohguro N, Sonoda KH, Takeuchi M (2012). The 2009 prospective multi-center epidemiologic survey of uveitis in Japan. Jpn J Ophthalmol.

[26] Polanía D, Reyes-Guanes J, Rojas-Carabali W (2022). A new look into uveitis in Colombia: changes in distribution patterns and clinical characteristics over the last 25 years. Graefes Arch Clin Exp Ophthalmol.

[27] Gritz DC, Wong IG (2004). Incidence and prevalence of uveitis in Northern California; the Northern California Epidemiology of Uveitis Study. Ophthalmology.

[28] Yang P, Zhong Z, Du L (2021). Prevalence and clinical features of systemic diseases in Chinese patients with uveitis. Br J Ophthalmol.

[29] Rathinam SR, Cunningham ET (2000). Infectious causes of uveitis in the developing world. Int Ophthalmol Clin.

[30] Kalogeropoulos CD, Stefaniotou MI, Gorgoli KE (2014). Ocular dirofilariasis: a case series of 8 patients. Middle East Afr J Ophthalmol.

[31] Khairallah M, Yahia SB, Ladjimi A (2007). Pattern of uveitis in a referral centre in Tunisia. North Africa Eye.

[32] de-la-Torre A, López-Castillo CA, Rueda JC (2009). Clinical patterns of uveitis in two ophthalmology centers in Bogota, Colombia. Clin Exp Ophthalmol.

[33] Islam SM, Tabbara KF (2002). Causes of uveitis at the eye center in Saudi Arabia: a retrospective review. Ophthalmic Epidemiol.

[34] Llorenç V, Mesquida M, Sainz de la Maza M (2015). Epidemiology of uveitis in a Western urban multiethnic population. The challenge of globalization. Acta Ophthalmol.

[35] Grajewski RS, Caramoy A, Frank KF (2015). Spectrum of uveitis in a German tertiary center: review of 474 consecutive patients. Ocul Immunol Inflamm.

[36] Engelhard SB, Patel V, Reddy AK (2015). Intermediate uveitis, posterior uveitis, and panuveitis in the Mid-Atlantic USA. Clin Ophthalmol.

[37] Wakabayashi T, Morimura Y, Miyamoto Y (2003). Changing patterns of intraocular inflammatory disease in Japan. Ocul Immunol Inflamm.

[38] McCannel CA, Holland GN, Helm CJ (1996). Causes of uveitis in the general practice of ophthalmology. UCLA Community Based Uveitis Study Group. Am J Ophthalmol.

[39] Biziorek B, Mackiewicz J, Zagorski Z (2001). Etiology of uveitis in rural and urban areas of mid-eastern Poland. Ann Agric Environ Med.

[40] Nalcacioglu-Yuksekkaya P, Ozdal PC, Yazici A (2015). Clinical and demographic characteristics of patients with uveitis starting later in life. Ocul Immunol Inflamm.

[41] Kalogeropoulos D, Anastasopoulos D, Gartzonika C (2017). Acute retinal necrosis in a patient with HSV-1 encephalitis. BAOJ Ophthalmol.

[42] Smit RL, Baarsma GS, de Vries J (1993). Classification of 750 consecutive uveitis patients in the Rotterdam Eye Hospital. Int Ophthalmol.

[43] Al Dhahri H, Al Rubaie K, Hemachandran S (2014). Patterns of uveitis in a university-based tertiary referral center in Riyadh, Saudi Arabia. Ocul Immunol Inflamm.

[44] Kotake S, Furudate N, Sasamoto Y (1997). Characteristics of endogenous uveitis in Hokkaido, Japan. Graefes Arch Clin Exp Ophthalmol.

[45] Cimino L, Aldigeri R, Salvarani C (2010). The causes of uveitis in a referral centre of Northern Italy. Int Ophthalmol.

[46] Weiner A, Ben ED (1991). Clinical patterns and associated conditions in chronic uveitis. Am J Ophthalmol.

[47] Çakar Özdal MP, Yazici A, Tüfek M (2014). Epidemiology of uveitis in a referral hospital in Turkey. Turk J Med Sci.

[48] Al-Shakarchi FI (2014). Pattern of uveitis at a referral center in Iraq. Middle East Afr J Ophthalmol.

[49] Asproudis I, Katsanos A, Kozeis N (2017). Update on the treatment of uveitis in patients with juvenile idiopathic arthritis: a review. Adv Ther.

[50] Birnbaum AD, French DD, Mirsaeidi M (2015). Sarcoidosis in the national veteran population: association of ocular inflammation and mortality. Ophthalmology.

[51] Laroni A, Calabrese M, Perini P (2006). Multiple sclerosis and autoimmune diseases: epidemiology and HLA- DR association in North-east Italy. J Neurol.

[52] Otasevic L, Zlatanovic G, Stanojevic-Paovic A (2007). Helicobacter pylori: an underestimated factor in acute anterior uveitis and spondyloarthropathies?. Ophthalmologica.

[53] Besada E, Schatz S, Saremi SS (2000). Post-streptococcal uveitis. Optometry.

[54] Cunningham ET, Forrester JV, Rao NA, Zierhut M (2016). Post-infectious uveitis. Ocul Immunol Inflamm.

[55] Muñoz Reyes MC, Romero Requena JM, Piña Alcántara YG, Bueno Álvarez-Arenas J, Ortiz CA (2019). Masquerade syndrome: an eye problem as a manifestation of a more sinister disease. Arch Soc Esp Oftalmol (Engl Ed).

[56] Rothova A, Groen F, Ten Berge JCEM (2021). Causes and clinical manifestations of masquerade syndromes in intraocular inflammatory diseases. Retina.

